# PDI-Functionalized Glass Beads: Efficient, Metal-Free Heterogeneous Photocatalysts Suitable for Flow Photochemistry

**DOI:** 10.1021/acs.oprd.4c00256

**Published:** 2024-09-06

**Authors:** Hamza Ali, Ifty Ahmed, Karen Robertson, Anabel E. Lanterna

**Affiliations:** † School of Chemistry, University of Nottingham, University Park, Nottingham NG7 2RD, U.K.; ‡ Advanced Materials Research Group, Faculty of Engineering, University of Nottingham, University Park, Nottingham NG7 2RD, U.K.

**Keywords:** heterogeneous photocatalaysis, sulfoxidation, continuous flow, process intensification

## Abstract

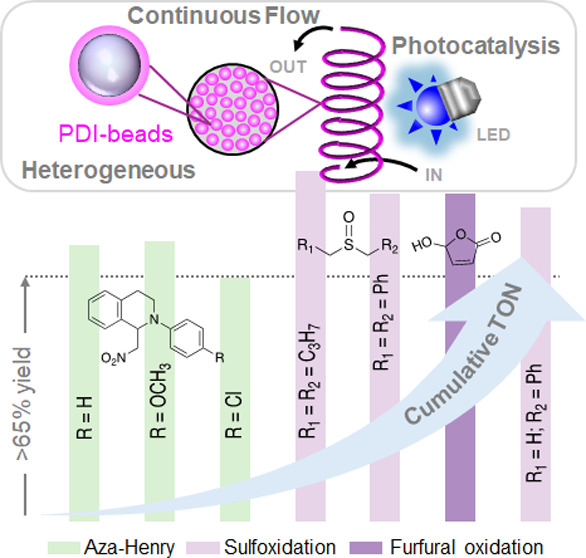

Perylene diimides (PDI) have an extraordinary ability to activate both energy and electron transfer processes upon light excitation; however, their extremely low solubility has hindered their wide use as photocatalysts. Here, we show that the combination of solid-supported PDIs with continuous flow photochemistry offers a promising strategy for process intensification and a scalable platform for heterogeneous photocatalysis. The photocatalyst immobilized onto glass beads is highly efficient, easy to separate, and extremely reusable, with a broad synthetic application range. Using the photo-oxidation of *n*-butyl sulfide as a benchmark reaction, we demonstrate that immobilized PDI are highly active, outperforming reported homogeneous photosensitizers, and capable of extensive reuse (turnover number (TON) >57,000 over 2 months). Transferring the process from batch to flow results in a 10-fold reduction in irradiation time and an increase in the space-time yield by a factor of 33 (40 vs 1338 mmol^–1^ h^–1^ L^–1^ batch vs flow). What is more, the same catalyst sample can be used for the preparation of a range of sulfoxides, the aza-Henry reaction between nitromethane and N–Ar tetrahydroisoquinolines, and the photo-oxidation of furfural with high catalytic activity. Overall, our work combines the remarkable photocatalytic properties of PDI with inert, easy-to-handle glass beads, producing hybrid materials that are reusable and can be adapted for performing heterogeneous photocatalysis in a range of scalable photochemical reactors.

## Introduction

Heterogeneous flow photocatalysis is an effective and efficient way to exploit the sustainable benefits of using light-to-power reactions, coupled with improved mass and heat transfer inherent to flow systems. Heterogeneous photocatalysts offer the potential for reusability and ease of removal from the reaction stream; however, their use in flow systems remains a challenge due to the inherent difficulty of flowing solids through narrow channels, causing issues such as clogging and poor flow dynamics.^1−4^ Many elegant designs of heterogeneous flow photoreactors such as trickle-bed,^5^ coated,^6^ suspension,^7^ spinning disk,^8^ parallel plate,^9^ and packed bed reactors (PBRs)^10^ aim to maximize contact with the heterogeneous catalyst surface, ensuring narrow residence time distribution (RTD) while working within safe operating pressures. Nevertheless, large-scale adoption of heterogeneous flow photocatalysis is limited in part due to the lack of efficient, reusable, and easy-to-separate photocatalysts.^11,12^

For a truly sustainable and versatile system, the catalyst needs to remain affixed and active over many cycles of reactions. This requires careful irradiation to avoid bleaching but also a strong attachment of the immobilized catalyst to prevent leaching. A common strategy to prepare heterogeneous photocatalysts relies on anchoring homogeneous photocatalysts onto silica-based supports such as glass wool,^13^ glass beads,^14^ silica gel,^15^ and mesoporous silica.^16^ Some of these examples^15^ rely on electrostatic interactions between the physisorbed catalytic species and the support, frequently resulting in catalyst desorption into the solution during use^17^ and making such materials unsuitable for flow chemistry applications. The formation of strong covalent bonds between the photocatalyst and support is necessary to prepare a durable, long-lived, and heterogeneous photocatalyst.^14,16,18,19^ Using this strategy, we have previously pioneered the use of inexpensive and widely available materials such as glass wool as supports of catalysts such as organometallic complex^13^ or metal nanoparticles^20^ suitable for use in batch and flow operation.^21^ Here, we present perylene diimides (PDIs) as suitable candidates for heterogeneous flow photocatalysis. PDIs are commonly used as photosensitizers^22−25^ due to their robust thermal and photochemical stability and their well-studied photoredox behavior. PDIs are known singlet oxygen (^1^O_2_) generators and have been used for a wide variety of organic reactions such as the reduction of aryl halides, iodoperfluorination of alkenes, and the aerobic oxidation of sulfides.^22,26^ Nevertheless, their wide use in homogeneous photocatalysis is hindered by their low solubility in common organic solvents. Attempts to use them in heterogeneous phase have been shown in the past,^19^ including use in combination with well-known inorganic photocatalysts such as TiO_2_.^27^ Nevertheless, the small particle size of commonly used supports presents issues when used with flow systems,^28^ as described above. In contrast, glass beads have good mechanical stability and can be fractioned via sieving, and their spherical shape permits ease of loading into flow reactors (via a slurry). They have been previously used as refractive components to increase light penetration^14,29^ and as static mixers within flow chemistry systems to improve mass transfer in multiphasic mixtures.^30^ As such, they are great candidates as supports for heterogeneous flow photocatalysis. In this study,^31^ we present the use of glass bead (solid and porous) supported PDIs as heterogeneous photocatalysts to carry out different organic transformations and suitable for use in batches and in a continuous flow-packed bed reactor.

## Results and Discussion

### Photocatalyst Preparation

**Figure 1:**
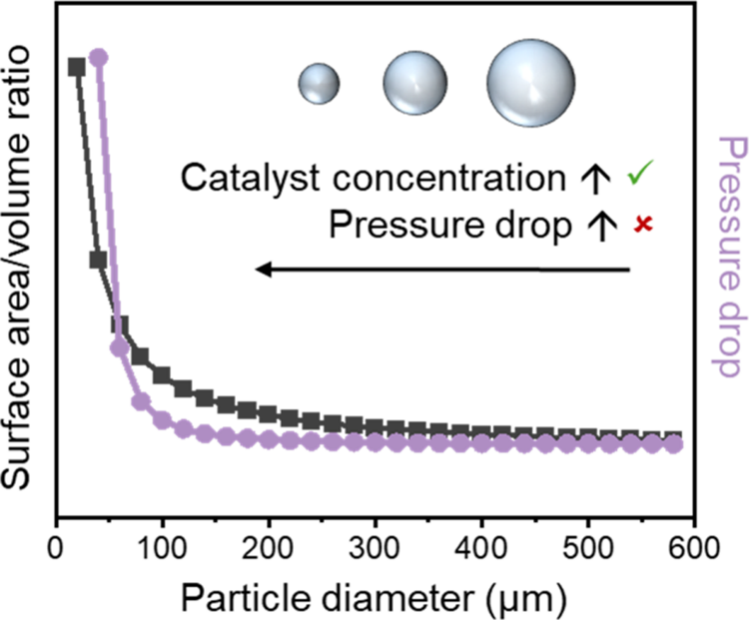
Plot showing (i) particle surface area/volume (SA/*V*) ratio and (ii) pressure drop as a function of particle diameter (*d*). Values were estimated considering SA/V is proportional to 1/*d*, and pressure drop is proportional to (1/*d*^2^ + 1/*d*), as per the Ergun equation (eq 1, SI).

**Figure 2:**
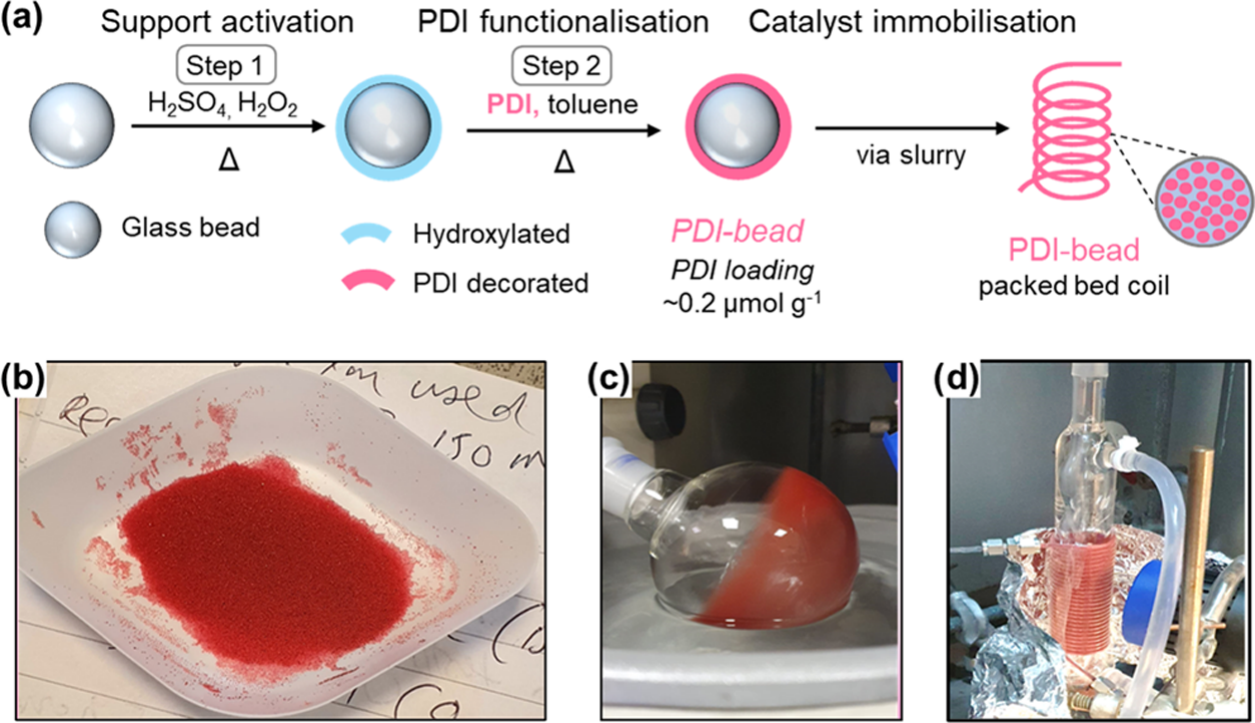
(a) Catalyst preparation showing the activation of the glass bead support followed by the covalent attachment of the PDI photocatalyst. The material is loaded into a fluorinated ethylene propylene (FEP) packed bed reactor via a slurry. (b) Photograph of glass beads functionalized with PDI. (c) Photograph of a round-bottom flask rotating in a rotary evaporator showing the PDI-beads forming a film on the walls of the flask. (d) Photograph of the FEP PBR loaded with PDI-beads.

**Scheme 1:**
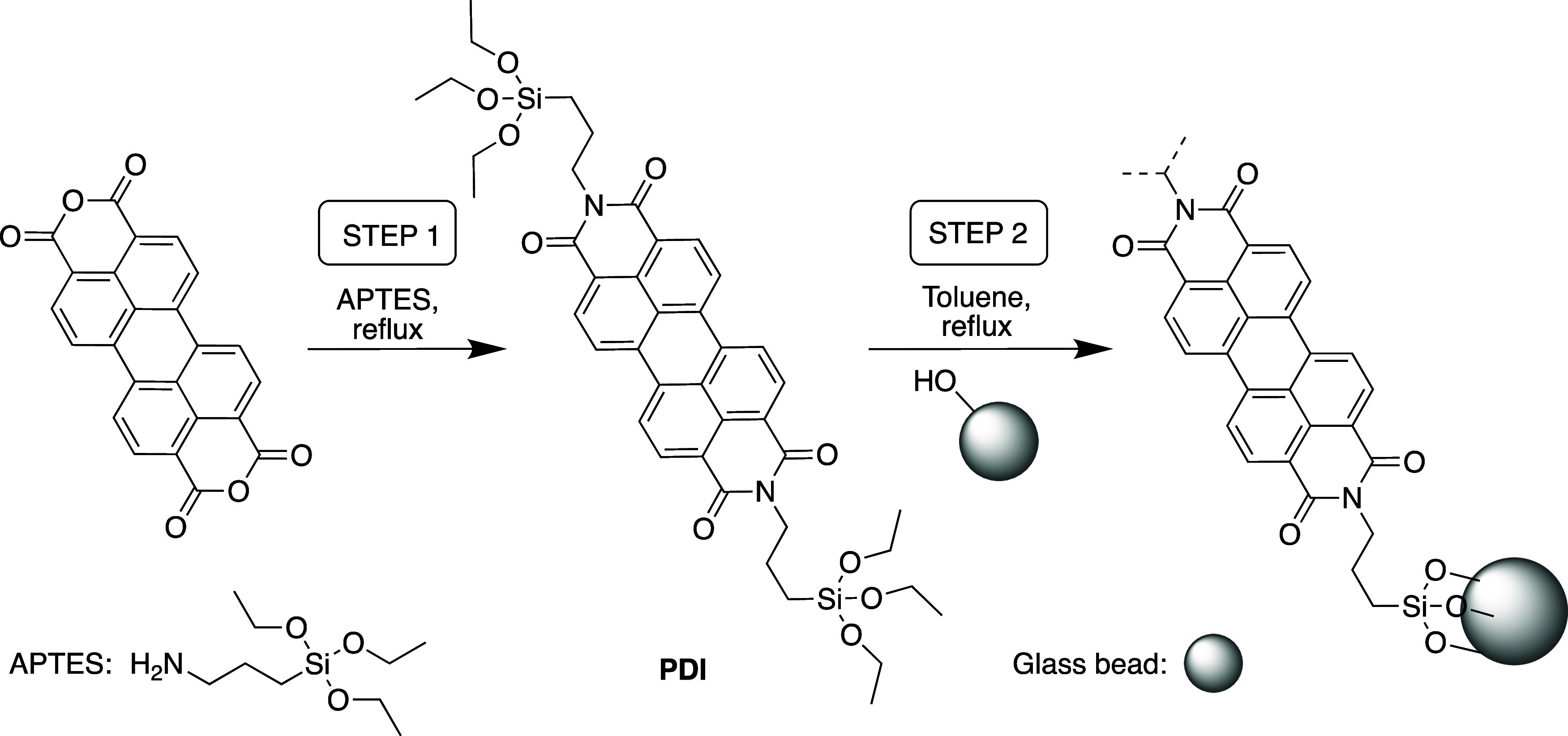
Immobilization Method for PDI-Supported Glass Beads: Step 1: Formation of the PDI Modified with 3-Aminopropyltriethoxysilane (APTES) in Dark Conditions (Aluminum Foil) under Inert Atmosphere; Step 2: Functionalization onto the Glass Bead Surface

Heterogeneous PDI photocatalysts are prepared using appropriately sized glass beads (see the SI); this includes solid glass beads (GB1) and bespoke^32^ porous phosphate-based glass beads PGB3 (Table S1) of particle size between 130 and 200 μm. These materials were deemed to have a size small enough to maintain a high surface-area-to-volume ratio for efficient catalyst loading while providing a safe range of operating pressures (Figures 1 and S1). The photocatalyst (PDI) was anchored onto the supports in a two-step process (Figure 2a). First, commercially available perylene-3,4,9,10-tetracarboxylic dianhydride (PTCDA) is functionalized with 3-aminopropyltriethoxysilane (APTES) to furnish the PDI shown in Scheme 1. Second, the APTES-ended PDI can be covalently attached via silanization onto preactivated commercial solid glass beads and custom porous microspheres through solution phase deposition without the need of coupling reagents. The resulting hybrid material shows a strong pink color, suggesting effective loading of the PDI onto the glass beads (Figure 2b). Figures S2–S7 show electronic microscopy images of the materials and spectroscopic characterization representative of the materials obtained using this protocol. The theoretical amount of PDI distributed across the surface of the glass bead was estimated by assuming glass bead geometry and formation of a PDI monolayer (Table S1). Experimental values were determined via PDI detachment upon base-catalyzed hydrolysis^33^ (see the SI) followed by analysis of the liberated dye using fluorescence spectroscopy analysis. This led to experimental loadings of 0.195 (±0.005) μmol g^–1^ for PDI-GB1, which is comparable to the theoretical value of 0.15 μmol g^–1^. The differences between predicted and measured loadings are likely due to deviations from theoretical assumptions such as microsphere size and the formation of a monolayer of PDI. In the case of porous beads (i.e., PDI–PGB3), the functionalization was performed without an activation step to avoid morphology changes seen when a pretreatment was used (Figure S5). Once the materials are prepared, the delivery of the catalyst into the reactors (round-bottom flask and PBR) is facile via a slurry (Figure 2).

### Photocatalytic Process: Batch Conditions

The suitability of the PDI-beads for heterogeneous photocatalysis was explored through the oxidation of *n-*butyl sulfide to *n-*butyl sulfoxide as a model reaction (Table 1). This reaction results in the insertion of a functional group commonly found within pharmaceuticals (e.g., omeprazole and sulindac^34^), agrochemicals, and polymers.^35,36^ Screening of the catalyst in the batch was completed in a thin-film rotary photoreactor inspired by the “Photovap” developed by George and Poliakoff^37^ using blue light irradiation (LEDs centered at 456 or 459 nm) as the excitation source to excite the PDI (see Figure S7). In this setup (Figure S8), the reaction proceeds to completion in less than 2 h when using MeCN or MeOH as solvents. The use of EtOH, a less toxic and greener solvent, shows slightly lower reaction kinetics despite theoretically having the greatest O_2_ solubility.^38^ Nevertheless, full conversion of the sulfide is achieved in 2.5 h with complete selectivity toward the sulfoxide. The presence of the overreaction product, i.e., *n-*butyl sulfone, was not detectable by gas chromatography (GC) or ^1^H NMR.

**Table 1:**
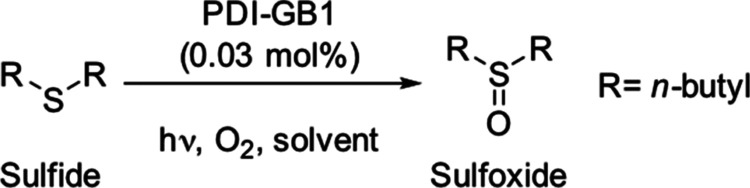
Solvent Effect on the Photocatalytic Oxidation of *n*-Butyl Sulfide under Batch Conditions^a^

entry	solvent	time (h)	conversion (%)	yield (%)
i	MeCN	1	72	68
ii	MeCN	2	>99	>99
iii	MeOH	1	68	63
iv	MeOH	2	>99	>99
v	EtOH	1	45	40
vi	EtOH	2	70	66
vii	EtOH	2.5	>99	>99

a Reaction conditions: 0.5 mmol *n*-butyl sulfide, 2 g PDI-GB1 (0.08 mol %), 5 mL O_2_-enriched solvent, O_2_ atmosphere. LED: 456 nm. Irradiance: 0.58 W cm^–2^. Reaction followed by gas chromatography (GC) using 1,3,5-trimethoxybenzene as an external standard.

**Figure 3:**
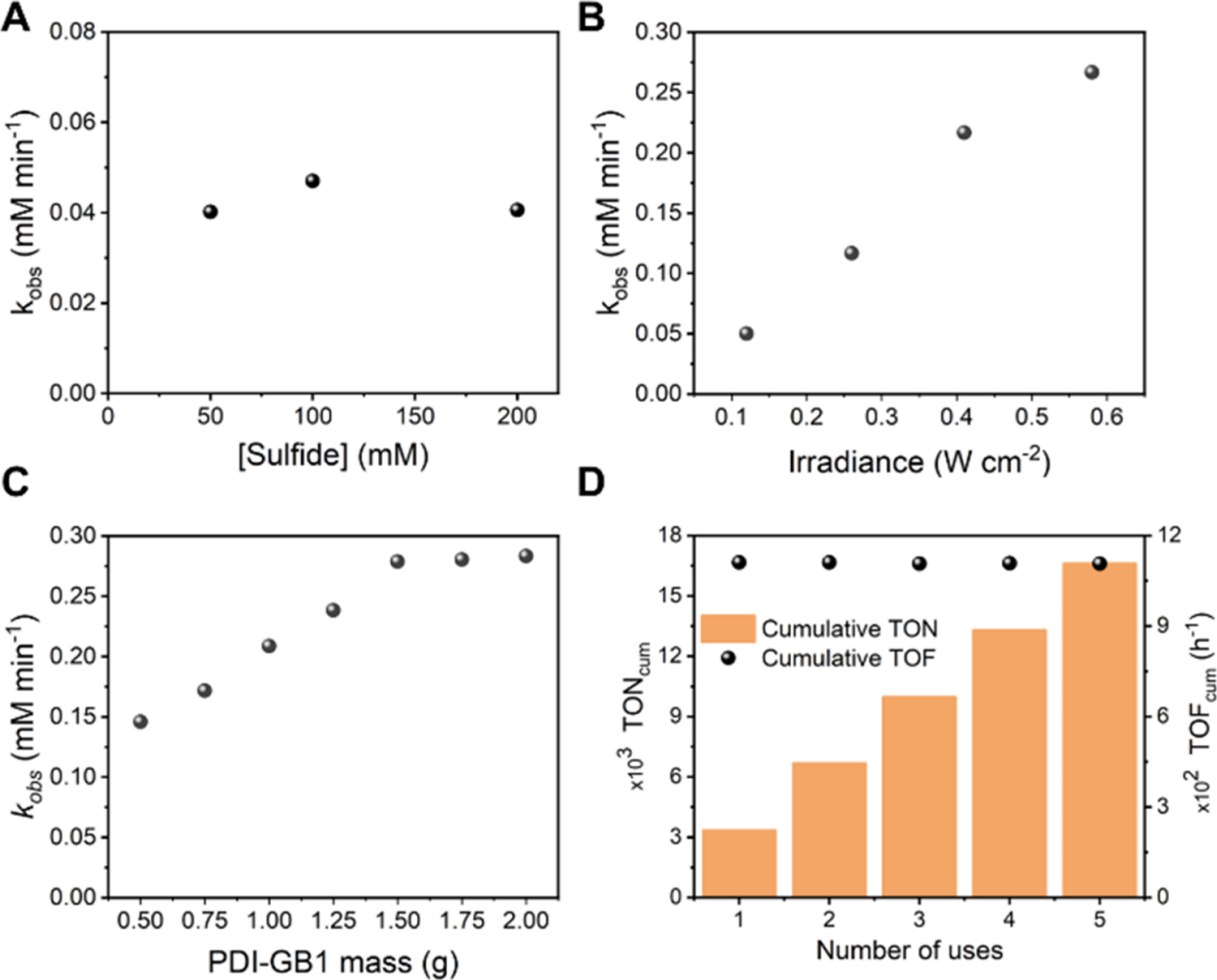
Kinetic studies to determine the order of reaction with respect to (A) *n-*butyl sulfide concentration (irradiance: 0.12 W cm^–2^), (B) light intensity, and (C) photocatalyst loading (MeOH instead of EtOH). (D) Reusability of PDI-GB1. Reaction conditions: 0.5 mmol *n-*butyl sulfide, 2 g PDI-GB1, 5 mL O_2_–enriched EtOH, O_2_ atmosphere. LED: 456 nm (typically 0.58 W cm^–2^). The reaction was followed by gas chromatography (GC) using 1,3,5-trimethoxybenzene as an external standard.

Control experiments (Table 2) show the reaction cannot proceed in the absence of a catalyst or in the dark (entries ii, iii). Also, both O_2_-enrichment of the solvent as well as maintaining an O_2_ environment within the flask were beneficial to increasing yield demonstrating the importance of maximizing O_2_ availability for the sulfoxidation to proceed. Kinetic studies show zero order with respect to the concentration of the substrate across the range explored (Figure 3A). Thus, the rate of the photo-oxidation step is limited by the generation of reactive oxygen species, which depends on light intensity, oxygen, and photocatalyst concentration. Figure 3B shows that the reaction rate increases linearly with light intensity. As commonly found in both homogeneous and heterogeneous photocatalytic systems, the reaction rate tends to increase linearly with photocatalyst concentration (Figure 3C) due to enhanced light absorption until reaching a plateau at peak light absorption. Scale-up of the reaction from 0.5 to 10 mmol of sulfide yields a 20-fold increase supplying quantitative reaction product in 16 h, comparable to similar aerobic oxidations of sulfides using homogeneous photosensitizers in batch.^39^ During these trials, the beads were reused 5 times without loss of activity, with a cumulative turnover number (TON) nearing 16,000 after the fifth run. The turnover frequency (TOF) remained largely unchanged across the five catalytic cycles (Figure 3D). Despite the retained catalytic activity, dye detachment was observed with roughly <1% of dye molecules lost over the five experiments (see Figure S10), potentially due to remnant PDI physisorbed via π–π bonding between PDI units. Furthermore, the shear mechanical forces experienced by the bead during rotation are expected to lead to attrition between particles and detachment of dye. These problems are inherent to any batch process, where mechanical mixing is necessary for effective mass transfer. As shown in the following sections, the passive mixing within the packed bed reactor (PBR) in combination with segmented (Taylor) gas–liquid premixing regime leads to excellent gas availability throughout the reactor, allowing for high reaction rates. Note that due to considerable differences between the two setups (modes of mixing, pressure, photon flux), the catalytic findings should not be directly compared.

**Table 2:** Oxygen Concentration Effect on the Photocatalytic Oxidation of *n-*Butyl Sulfide under Batch Conditions^a^

entry	change of conditions	conv (%)	yield (%)
i	none	>99	>99
ii	dark	trace	0
iii	no catalyst	trace	0
iv	O_2_-enrichment, air atm	28	25
v	no O_2_-enrichment, O_2_ atm	51	49
vi	O_2_-enrichment, Ar atm	11	8
vii	Ar degassing, Ar atm	trace	0
viii	Ar degassing, O_2_ atm	45	43

a Reaction conditions: 0.5 mmol *n*-butyl sulfide, 2 g PDI-GB1 (0.08 mol %), 5 mL EtOH, 2 h irradiation. LED: 456 nm. Irradiance: 0.58 W cm^–2^. Reaction followed using gas chromatography (GC) with 1,3,5-trimethoxybenzene as an external standard.

### Photocatalytic Process: Flow Conditions

Typically, flow experiments using heterogeneous photocatalysts are performed by flowing the catalysts in slurries.^40,41^ Under these conditions, attrition and leaching can still be a problem. In contrast, PBRs retain the catalyst in the tube while the reagents are flown through.^42^ The benefits of this approach include ease of assembly, effective mixing between phases, and immobilization of the catalyst, leading to simpler separation and avoiding wasteful catalyst removal steps.^43^ As mentioned above, we evaluated the suitability of different supports to be deployed in PBRs. For this, a series of pressure drop experiments were conducted on the flow reactor with silica gel, commercial glass beads (GBx), and the custom porous microspheres (PGBx; see the SI for details) to determine their appropriateness as supports. Silica gel was excluded due to the high back pressures experienced, while the glass microspheres performed well with a ∼20% reduction in pressure drop for PGB3 compared to GB1.

The full reactor setup can be found in the SI (Figure S9); briefly, the liquid and gas streams combine at a T-piece to provide segmented flow prior to the PBR (0.085” ID). This is illuminated with a 459 nm LED (1.2 W cm^–2^) lamp and cooled from an internal cooling column run from a water circulator.

Initially, we explored short PBR (10 cm long) for solid and porous supports (Table 3 entries i–iv). The solid glass beads (PDI-GB1) were found to perform with excellent efficiency (>99% conversion to the sulfoxide). Under the same conditions, the porous beads (PDI-GB3) had a 32% reduction in conversion (67% overall conversion) and around 20% formation of the corresponding sulfone (R_2_SO_2_). This is potentially due to the longer residence of products within the micropores of PGB3, thereby increasing time under illumination. To support this hypothesis, we evaluated residence time distributions (RTD) for PDI-GB1 and PDI–PGB3 under conditions described in SI. The results show slightly different RTD profiles (Figure S12). The solid glass beads have a narrower distribution compared with the porous glass microspheres. The broader RTD profile in combination with a longer mean residence time is thought to be the cause of the reduced conversion and increased formation of *n-*butyl sulfone, likely due to the presence of porous supports like PGB3.^44^

**Table 3:** Flow Syntheses of *n-*Butyl Sulfide Completed in a Packed Bed Photochemical Reactor^a^

								productivity	
entry	catalyst	[sulfide] (mM)	*Q* (mL min^–1^)	O_2_ (mL min^–1^)	τ_res_ (min)	conversion (%)	yield (%)	mmol h^–1^	mmol h^–1^ L^–1^
i^b^	PDI-GB1	25	0.025	0.01	4.2	78	74	0.04	265
ii^b^	PDI-GB1	25	0.010	0.01	7.3	>99	96	0.03	197
iii^b^,^c^	PDI–PGB3	25	0.025	0.01	4.2	60	40	0.02	143
iv^b^,^c^	PDI–PGB3	25	0.010	0.01	7.3	67	52	0.02	107
v^d^,^e^	PDI-GB1	50	0.4	0.20^e^	3.7	>99	96	1.73	787
vi^d^,^e^	PDI-GB1	75	0.4	0.20^e^	3.7	>99	95	2.57	1168
vii^d^,^e^	PDI-GB1	100	0.4	0.20^e^	3.7	68	60	2.16	983
viii^d^,^e^	PDI-GB1	100	0.2	0.20^e^	5.5	>99	>99	2.38	1082
ix^d^,^e^	PDI-GB1	100	0.3	0.20^e^	4.4	>99	98	2.94	1338

a Reaction conditions: *n*-butyl sulfide, O_2_-enriched EtOH. LED: 459 nm working at 1.2 W cm^–2^. Reaction followed using GC-FID with 1,3,5-trimethoxybenzene as an external standard.

b Reactor length (*L*): 10 cm with 10 cm of 0.085” ID tubing for prereaction gas–liquid segmentation. Reactor volume: ∼150 μL.

c Yield drops are the result of ∼20% sulfone formation.

d Reactor length (*L*): 150 cm with 10 cm of 0.085” ID tubing for prereaction gas–liquid segmentation. Reactor volume: ∼2.2 mL.

e O_2_ flow rate: 0.16–0.24 mL min^–1^, average: 0.20 mL min^–1^. *Q*: liquid flow rate, *t*_res_: estimated space time assuming 40% void fraction for both porous and solid beads, differences in conversion could be a result of differing photon equivalents received by each catalyst during the reaction. Productivity (mmol h^–1^) = concentration (*M*) × flow rate (mL min^–1^) × yield × 60 min h^–1^. Space-time yield (STY, mmol h^–1^ L^–1^) = productivity/reactor volume.

Gratifyingly, no PDI leaching was detected in the reaction mixture under flow conditions, as opposed to the loss of PDI experienced in the rotary thin-film reactor (Figure S11). This suggests that in continuous flow operation, the material experiences lower shear forces than in batch, resulting in low mechanical stress and undetectable catalyst loss (via UV–vis, fluorescence, or NMR spectroscopy analyses). Furthermore, small particles of borosilicate glass carried forward from the commercial manufacturing process and surface activation can readily detach in batch due to the rotary nature of the reactor but are unlikely to do so in continuous flow. Any potential (undetected) detachment of fine solids is confined within the reactor due to glass wool plugs on the reactor inlet/outlet. The back pressure generated during fluid flow at process conditions was typically less than 10–15 psig, which provides confidence in the scale-up of this reactor through either a longer coil or a wider diameter tubing. Furthermore, typical issues experienced when using heterogeneous materials in flow such as blocking and clogging were not experienced due to low back pressure generated by the ca. 200 μm catalyst support.

### Productivity Increase

To increase productivity, longer reactor lengths (150 and 300 cm) were used for faster reagent flow. The concentration of *n-*butyl sulfide could be increased from 50 to 75 mM, leading to an increase in hourly productivity from 1.73 to 2.57 mmol h^–1^ (Table 3, entries v–vi). An attempt was made to maintain the same estimated residence time (*t*_res_: 3.7 min) and further increase the reaction concentration to 0.1 M, but this led to poorer conversion and a yield of 60% (Table 3, entry vii). Nevertheless, increasing the residence time to 5.5 min achieved quantitative conversion into *n-*butyl sulfoxide, with an hourly productivity of 2.38 mmol h^–1^ (Table 3, entry viii). The effect of flow rate was explored further, and it was possible to maintain a comparable yield with a *t*_res_ of 4.4 min, increasing the hourly productivity to 2.94 mmol h^–1^ (11.4 g day^–1^, Table 3, entry ix). A longer reactor (300 cm long) can be assembled, keeping a similar productivity (see Table 4, entry (i)).

**Table 4:** Performance of the Heterogeneous Photocatalytic Flow System Reusing the Same Photocatalyst (PDI-GB1) under Scaled-Up Conditions and Using Three Different Oxidation Reactions^a^

								productivity	
entry	*R*_1_	*R*_2_	cumulative TON	*Q* (mL min^–1^)	O_2_ (mL min^–1^)	τ_res_ (min)	yield (%)	(mmol h^–1^)	(mmol h^–1^ L^–1^)
Oxidation of Sulfides^b^									
i	*n*-Bu	*n*-Bu	13,000	0.098	0.195	16	95	3.34	732
ii	Bn	Bn	19,528	0.073	0.147	21	89	2.35	515
iii^e^	Ph	Me	57,089	0.073	0.147	21	85	2.24	492
Oxidative Aza-Henry Reaction^c^									
iv	H	N/A	2125	0.037	0.111	31	76	0.51	111
v	OMe	N/A	4208	0.037	0.111	31	75	0.50	110
vi	Cl	N/A	6042	0.037	0.111	31	66	0.44	96
Oxidation of Furfural^d^									
vii^f^	N/A	N/A	48,783	0.100	0.200	15	89	3.2	702

a Reaction conditions: LED: 456 nm working at 0.58 W cm^–2^. Reactor length (*L*): 300 cm with 10 cm 1/16” ID presegmentation tubing. Reactor volume: 4.4 mL. Catalyst: ∼30 g of PDI-beads. *Q*: liquid flow rate, τ_res_: estimated residence time assuming 40% void fraction. Productivity (mmol h^–1^) = concentration (*M*) × flow rate (mL min^–1^) × yield × 60 min h^–1^. Space-time yield (STY, mmol h^–1^ L^–1^) = productivity/reactor volume.

b Sulfide (0.2 M) in O_2_-enriched EtOH. Reaction followed using gas chromatography (GC) with 1,3,5-trimethoxybenzene as an external standard.

c N–Ar THIQ (0.075 M) in 4:1 MeCN/MeNO_2_ solvent. Reaction followed using ^1^H NMR with 2,5-dimethylfuran as an external standard.

d Furfural (0.2 M) in MeOH, *p*-toluenesulfonic acid (0.05 mol %). Reaction followed using GC with 1,3,5-trimethoxybenzene as an external standard.

e This entry corresponds to the last catalytic cycle run with the same PDI-GB1 sample; postreaction analysis suggests 0.120 μmol g^–1^ PDI still attached onto the beads (from initial 0.195 μmol g^–1^). Catalytic activity is still remarkable.

f –60 cm 1/32” ID segmentation tubing instead of 10 cm 1/16” ID. Note that with increased reactor size, a greater proportion of the reactor relies on reflected light for photoexcitation, and this might be reflected on the similar conversions observed between entries iv–vi and those seen in Table S3.

### Substrate Scope

**Scheme 2:**
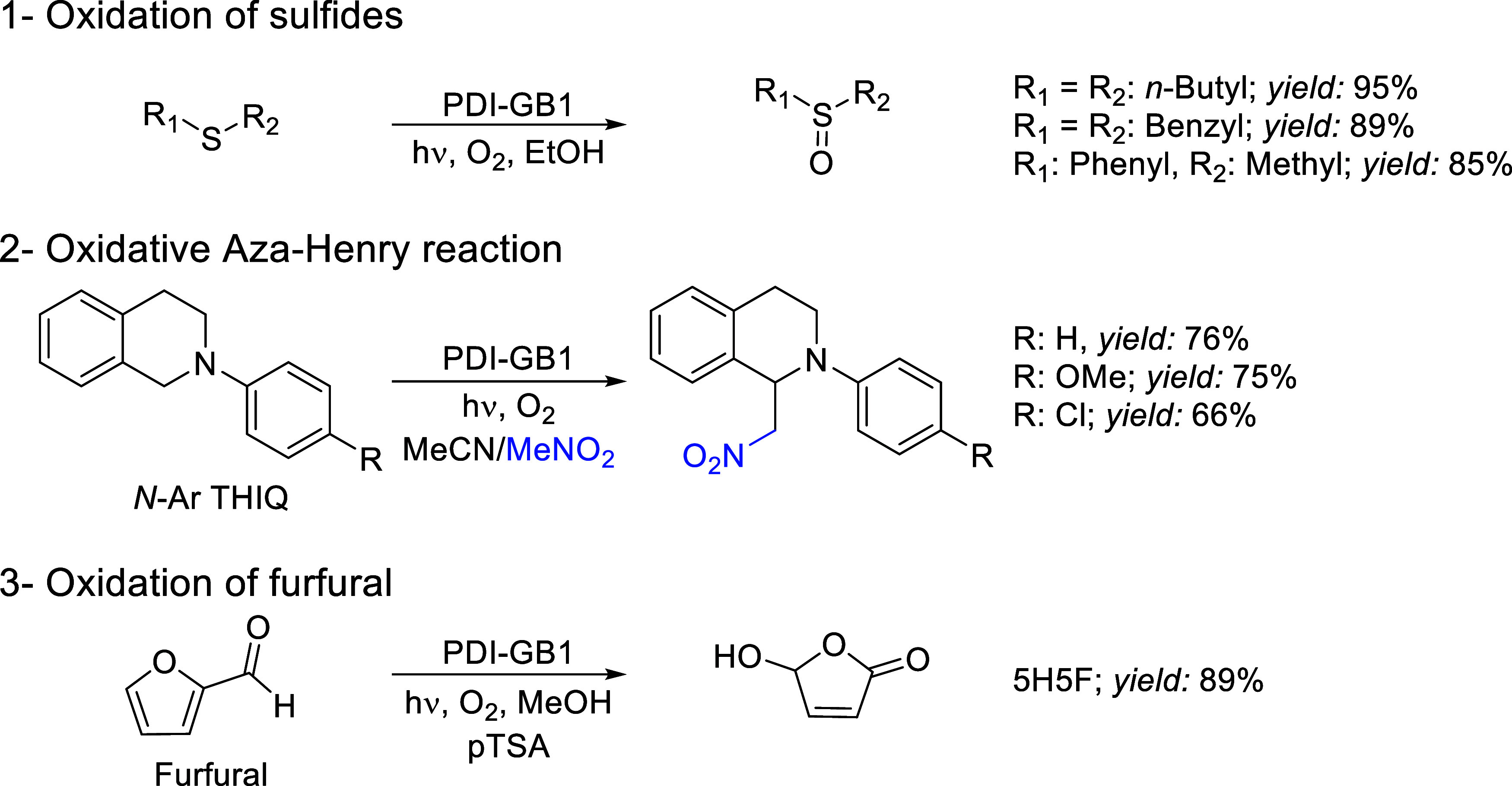
Oxidation Reactions Performed in the Heterogeneous Photocatalytic Flow System Reusing the Same Photocatalyst (PDI-GB1) under Scaled-Up Conditions (See Table 4)

The versatility of this heterogeneous photocatalytic flow system was tested using other sulfides (thioanisole and dibenzyl sulfide) as well as applied to different oxidation reactions, such as the aza-Henry reaction and the oxidation of furfural to 5-hydroxy-2(*5H*)-furanone (*5H5F*) shown in Scheme 2 and Figure 3. Remarkably, all reactions were completed using the same catalyst sample (Table 4, cumulative TON column), starting with the oxidative aza-Henry reaction, followed by the aerobic oxidation of sulfides and furfural, respectively. Particularly, the protocol for the oxidation of sulfides was extended to dibenzyl sulfide with good yield, resulting in a projected daily productivity of 13 g day^–1^ (Table 4, entry ii). Thioanisole was converted to the corresponding methyl phenyl sulfoxide in 85% yield, demonstrating the applicability of this protocol to alkyl/aryl containing sulfides.

Additionally, the visible light promoted aza-Henry reaction, commonly used to assess the efficiency of heterogeneous photoredox catalysts,^45−47^ was successfully performed by our photocatalytic system. The transformation results in a C–C coupling between *N*-aryl tetrahydroisoquinoline (N–Ar THIQ) and a nucleophilic coupling partner such as nitromethane (Tables 4 and S3). The reaction proceeded with high conversion and selectivity toward the coupled product (0.51 mmol h^–1^, 111 mmol h^–1^ L^–1^). Functional analogues containing an electron-withdrawing or an electron-donating group on the aryl substituent were also successfully converted with productivities of 0.44–0.5 mmol h^–1^ and a projected space-time yield of 96–110 mmol h^–1^ L^–1^. Finally, we evaluated the oxidation of furfural, a bioderived feedstock with great potential to produce value-added chemicals (VACs).^48^ This is the oxidation derivative of furfural, *5H5F*, which can be further processed into a wide range of VACs such as plastic monomers^49^ and C4 building blocks such as 1,4-butanediol and pyrrolidones,^50^ providing an alternative route toward petroleum-derived chemical products. The light-driven production of *5H5F* is typically completed with homogeneous organic dyes such as rose bengal and methylene blue.^51^ On an industrial scale, considerations around the environmental footprint of the process are needed due to the single use nature of such catalysts as well as the cost (solvent, auxiliaries) associated with their removal.^52^ Here, we achieved oxidation of furfural to *5H5F* with 89% yield and no alkoxylated product (5-methoxy-2(*5H*)-furanone) detected (Tables 4 and S4). Addition of catalytic amounts of *p-*toluenesulfonic acid (pTSA) dramatically increased the rate of reaction, thought to be due to the formation of a more reactive furfural dimethyl acetal,^53^ although at the cost of an additional workup step. The coupling of a visible light acetalization process with this photo-oxidation protocol could potentially allow for a high throughput telescoped synthesis of *5H5F* and further derivatives, without the need for additives or purification of the reaction mixture.^54^

## Conclusions

**Figure 4:**
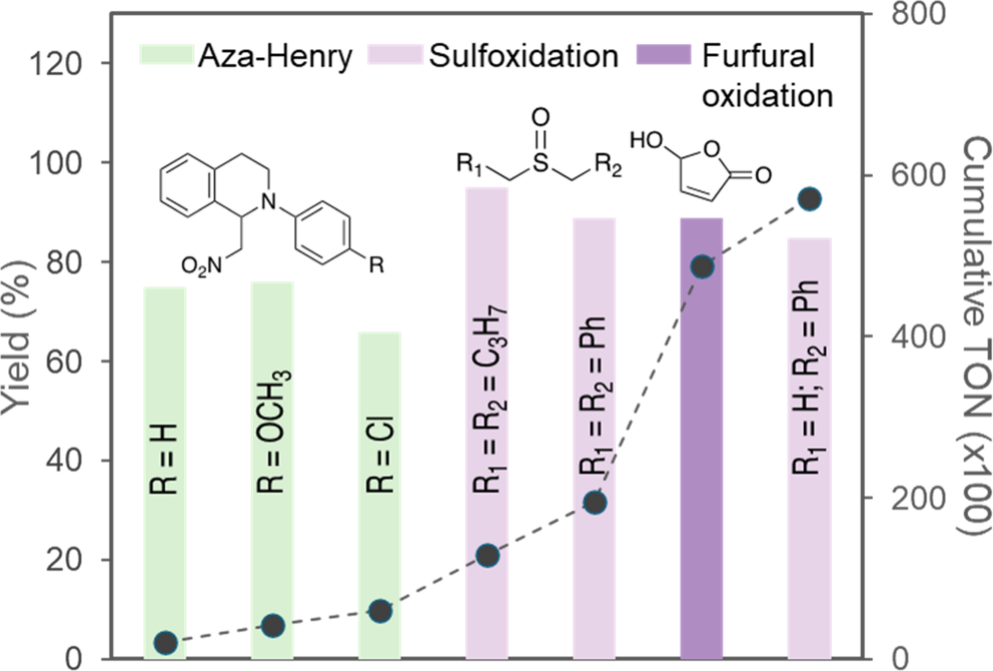
Scope of reactions performed in a continuous flow PDI-bead packed bed reactor using the same sample of PDI-beads catalyst. The reactions are shown from left to right in the order they were performed. Bars show yields of target products, and dots show cumulative TON. Reaction conditions are shown in Table 4.

We used a glass-supported perylene diimide heterogeneous photocatalyst (PDI-beads) for continuous flow synthesis of a range of compounds at the decagram scale. Benchmarking of the materials with oxidation of *n-*butyl sulfide showed fast kinetics in batch, which translated into high performance in flow (1338 mmol h^–1^ L^–1^). Our system is superior with respect to common heterogeneous photocatalysts such as carbon nitrides,^55^ which require an aldehyde radical initiator to promote the oxidation of sulfides, generating further chemical waste. The support type was optimized and scale-up achieved through an increased reactor length and flow rate, resulting in a simple continuous flow synthesis platform. Exploration of substrate scope encompassing the aza-Henry reaction and oxidation of furfural with a total of seven different chemical targets produced good yields and high productivity (Figure 4). Whereas other reported oxidations of sulfides (1 mol day^–1^) have safety concerns as they work with pressurized O_2_ (8 barg) and become more costly as they require homogeneous photocatalysts,^56^ our system works with atmospheric pressures of O_2_, and the same sample of catalyst was used throughout the substrate scope exploration amounting to a total cumulative TON of >57,000, with no changes in productivity. The use of PDI-beads in a simple FEP PBR intensifies the synthetic process due to concurrent catalysis and separation, eliminating the need for a costly and wasteful catalyst removal stage and opening the possibility of telescoped chemical synthesis. As catalyst cost and removal or recycling are the major economic and environmental concerns in process chemistry,^57^ the rapid reuse of our catalyst and no extraction method required between reactions due to the heterogeneous nature of the PDI-beads present a highly versatile and sustainable option for future photocatalytic applications.

## Experimental Section

### Materials and Methods

Commercially available solid glass beads (150–212 μm, 500–750 μm) as well as a range of porous, phosphate-based glass microspheres were used as supports. Hydrogen peroxide (12%) and sulfuric acid (S.G.1.8) were used to prepare a piranha solution for support surface activation. Perylene-3,4,9,10-tetracarboxylic dianhydride (PTCDA) and 3-aminopropyltriethoxysilane (APTES) were used to prepare the PDI. Analytical standards *n-*butyl sulfide, *n-*butyl sulfoxide, *n-*butyl sulfone, and 1,3,5-trimethoxybenzene were purchased from Sigma-Aldrich. FEP tubing was purchased from Cole-Parmer. Fittings for the flow reactor were obtained from Swagelok and Cole-Parmer. Two different lamps were used in this study: a Kessil PR160L (456 nm, 0.58 W cm^–2^) and a custom-made LED block (459 nm, 1.2 W cm^–2^) designed at the University of Nottingham.

### Synthetic Procedures

#### Synthesis of PDI

PDI was synthesized via a reported literature method^19^ with slight modifications. Briefly, PTCDA (2 g) was purged with N_2_ in a round-bottom flask. APTES (4 mL, ∼4 equiv) was injected into the flask, and the resulting mixture was stirred for 20 h under an N_2_ atmosphere in the dark at 150 °C. The resulting red solid was ground to a fine powder and transferred to a Soxhlet system for extraction with petroleum ether for 7 days to remove unreacted APTES, followed by extraction with acetone for 2 days to remove residual PTCDA. Acetone was used to recover the solid and subsequently removed under reduced pressure to afford the PDI as a dark red powder (87%).

#### Activation of Support

The surface of the glass beads was activated using Piranha solution (3:1 H_2_SO_4_:H_2_O_2_), washed with DI water, and dried in an oven at 80 °C for 2 h. For porous beads (PGB1–3), a concentrated base etch (NaOH, 10 M, methanolic) was used at room temperature.

#### Synthesis of PDI-Beads

PDI was immobilized onto silica supports by using a standard protocol (Scheme 1). Depending on the nature of the support, one of two activation routes was taken (above). The activated beads were immediately transferred into a round-bottom flask with 50 mL anhydrous toluene and 0.3 g of PDI and refluxed overnight. The PDI-beads were washed with toluene (x2), acetone (x2), and methanol to remove unreacted PDI and transferred to a Soxhlet system for extraction with acetone for 2 days.

### Photocatalytic Procedures

#### Batch Conditions

The batch photochemical oxidation of *n-*butyl sulfide was carried out in a thin-film rotary photoreactor (Figure S8). Typically, a 50 mL round-bottom flask was charged with 0.5 mmol *n*-butyl sulfide, 5 mL O_2_-enriched solvent, and 2 g PDI-beads. The flask was connected to the evaporator under an oxygen environment. Rotation speed was set to simultaneously suspend the glass beads as well as create a thin film to maximize the contact between gas–liquid–solid phases. The flask was partly submerged in a water bath (to maintain a constant reaction temperature between 18 and 20 °C) and irradiated using an LED lamp. For the gram scale experiment, the reaction was completed in a 250 mL round-bottom flask with 15 g PDI-beads in 10 mL oxygen-enriched EtOH (obtained after bubbling O_2_ through the solvent for 10 min), irradiating with blue LED (0.58 W cm^–2^) for 16 h. Reactions were followed by gas chromatography (GC) using 1,3,5-trimethoxybenzene as an external standard.

#### Flow Conditions

Flow photochemistry was tested in a packed bed flow reactor (Figures S8 and S9). A flow photoreactor was prepared by loading a fixed mass of PDI-beads into FEP tubing (0.125” OD, 0.085” ID) and wrapping this coil around a condenser to allow for cooling and effective irradiation. 30 g PDI-beads was typically required to prepare a 3 m long PBR. The irradiation sources were placed 2.5 cm from the surface of the flow reactor. A syringe was loaded with a solution of the substrate in an O_2_-enriched solvent. Liquid flow into the system was controlled using an HPLC pump (Varian Prostar) or syringe pump (World Precision Instruments) and was combined with a stream of O_2_ using a T-mixer. Gas flow was metered into the reactor by using a mass flow controller (Alicat Scientific, Inc.) The two fluid streams were combined in a T-mixer until a segmented flow pattern was achieved. Temperature was maintained at 20–30 °C in the reactor through water cooling of the coil holder. The reactor was irradiated using a blue LED lamp, and analytical samples were acquired after 3 reactor volumes to measure steady-state concentration.

#### Safety Recommendations

The use of gaseous oxygen within chemical reactors poses safety concerns due to the flammability of many organic solvents. To reduce the risk of operating under these conditions, reactions must be completed in well-ventilated fumehoods, clean of any organic contaminants or grease. The delivery of gas can be facilitated through a mass flow controller and a regulated gas cylinder, allowing for small amounts of gas to be introduced and avoiding accumulation of gas and formation of a headspace. It is important to highlight that all of the experiments are performed at atmospheric pressure.

#### Characterization

Gas chromatography (GC) was completed on a Thermo Fisher 1310 system coupled with a flame ionization detector (FID). FT-IR spectra were acquired by using a Bruker α IR spectrometer with an ATR accessory. A JEOL 6490LV scanning electron microscope was used to evaluate the size and morphological properties of the solid-state materials used in this study. Absorption properties of the synthesized materials were evaluated using an Agilent Cary 5000 spectrophotometer using a DRA-1800 (PMT/InGaAs) diffuse reflectance accessory. Absorption and emission spectroscopies were obtained by using a Tecan Infinite 200 PRO plate reader. Solid-state photoluminescence spectra were acquired using an Edinburgh Instruments FLS980 system using an Xe source lamp and a 590 nm cutoff filter.

Further experimental details and data are provided in the accompanying Supporting Information.

## Supporting Information Available

Further experimental details about catalyst preparation and characterization, photochemical reactors, reactions optimization, residence time distribution analysis, and turnover number calculations (PDF)
