# Supplementary material for: PDI-Functionalized Glass Beads: Efficient, Metal-Free Heterogeneous Photocatalysts Suitable for Flow Photochemistry
Source: Org Process Res Dev. 2024 Sep 6;28(9):3698–706. doi: 10.1021/acs.oprd.4c00256 (PMC11421094; doi:10.1021/acs.oprd.4c00256)
Supplement: Supplementary file 1 — op4c00256_si_001.pdf [file op4c00256_si_001.pdf]

Supporting information for

PDI-functionalized glass beads: efficient, metal-free heterogeneous photocatalysts suitable for flow photochemistry

Hamza Ali[a,b], Ifty Ahmed[b], Karen Robertson*[b] and Anabel E. Lanterna*[a]

[a]School of Chemistry, University of Nottingham, Nottingham, NG7 2RD, UK.

[b]Advanced Materials Research Group, Faculty of Engineering, University of Nottingham, Nottingham, NG7 2RD, UK.

Table of Contents

Experimental details 4

Support selection 4

Table S1. Properties of the catalyst support materials used in this work. 4

Figure S1. Back pressure experienced as a function of IPA flow rate over a fixed-bed reactor (ID = 0.085 in) for amorphous silica gel, GB1, PGB1, PGB2 and PGB3. The plots compare particle morphology and particle porosity (A), and porous particle size (B). RSD < 1 %. 5

Pressure drop calculations 5

Pressure drop experiments 6

Materials characterization 7

Figure S2. Representative scanning electron microscopy images of A) GB1, B) GB2, C) PGB1, D) PGB2, E) PGB3, and F) silica gel for comparison. 7

Figure S3. Low magnification SEM image of GB1 (A) and the corresponding particle size distribution (measured using ImageJ) (B). 8

Figure S4. Low magnification SEM images of A) PGB1, C) PGB2, and E) PGB3; and the corresponding particle size distributions B), D), and F). Photocatalyst preparation 9

Figure S5. A) Low magnification (x150) SEM image of PDI-PGB3 after functionalisation showing some rupture structures, B) low magnification (x80) SEM image of PDI-PGB3 after functionalisation without an activation stage showing porous structure is retained, and C) medium magnification (x500) SEM image of PDI-PGB3 showing the presence of micron-sized pores and wall-to-wall porosity. 10

Figure S6. ATR FTIR spectra comparing PTCDA (pink), APTES (amber) and PDI (purple). 11

Figure S7. Optical characterisation of PDI-beads: diffuse reflectance (DR) spectra of A) PDI-GB1 and B) PDI-PGB3 beads prepared without activation (largely intact, Figure S5B).. 12

PDI loading.. 13

Scheme S1: Hydrolysis of silane from support surface.. 13

Table S2. Theoretical PDI loading.. 13

Photochemical reactors 14

Figure S8. Schematic representation of the photoreactors used in this work (adapted from ref [8]). 14

Figure S9. Complete schematic representation of the flow system used in this work, as shown in the scheme the reactor is vertically oriented and the fluids are flown from the bottom of the coil upwards at a slight pitch (corresponding to the pitch of the coil wrapped around the condenser). 15

Figure S10. Absorption spectra recorded from each catalytic cycle run under batch conditions. Considering PDI extinction coefficients are typically in the tens of thousands (~40,000 $M^{-1}cm^{-1}$),[10] the estimated amount of PDI loss is <1% of the total PDI loading (assuming a PDI loading of 0.195 µmol g^{-1}). Note the leaching could be due to formation of multiple layers or covalently bound PDI detaching due to particle attrition resulting from mechanical mixing. 16

Figure S11. Absorption spectrum recorded from a reaction run under flow conditions. No PDI loss was detected under absorbance (blue) or fluorescence spectroscopy (not shown). The peak centred at ~300 nm corresponds to n-butyl sulfoxide (reaction product). 17

Residence time distribution analysis... 17

Figure S12. Residence time distribution of a pulse injection through packed beds of PDI-GB1 (red) with mean residence time = 17.8 min and PDI-PGB3 (black) with mean residence time = 28.4 min when using a flow rate of 50 µL min^{-1}. 18

Turnover number calculations 19

Table S3. Optimisation of flow conditions for photocatalysed Aza-Henry reaction between N-Ph THIQ and nitromethane... 19

Table S4. Optimisation of flow conditions for photo-oxidation of furfural to 5H5F 20

References... 21

Experimental details

Support selection

Glass bead supports (Table S1) with different sizes and morphologies were evaluated: from commercial glass beads (GB1, entries i) to porous phosphate-glass microspheres (PGB1, PGB2, and PGB3; entries ii-iv) manufactured in-house at the University of Nottingham. The materials were selected to provide varied surface area for catalyst loading while reducing pressure drops when used in fixed-bed flow reactors[1-4]. All materials were characterised by scanning electron microscopy (see below).

Table S1. Properties of the catalyst support materials used in this work.

Entry	Label	Composition	Morphology	Particle size (μm)[a]
i	GB1	Borosilicate glass[b]	Solid microspheres	133 – 215
ii	PGB1	Phosphate glass [c,d]	Porous microspheres	65 – 124
iii	PGB2	Phosphate glass [c,d]	Porous microspheres	115 – 206
iv	PGB3	Ti-doped phosphate glass[c,d]	Porous microspheres	118 – 211

[a] Determined by SEM imaging analysis. [b] Commercially available. [c] Materials synthesised following reported methods[5]. [d] Materials are soluble at low pH aqueous solutions.

To test the glass beads suitability for a fixed-bed reactor (Figure S8), we designed a series of pressure drop experiments to determine the correlation between particle morphology and pressure generation under fluid flow conditions. Generally, pressure drop per unit length ($\Delta P/L$) increases[6] with decreasing particle size and increasing packing density. Thus, the ideal support should be capable of being deployed in a fixed-bed reactor without being too fine to generate high pressures, yet small enough to maintain a high surface area-to-volume ratio for efficient catalyst loading. Figure S1 shows the pressure drops experienced in fixed-bed reactors related to particle size, porosity, and reactor dimensions. As expected, the support morphology plays a crucial role in the pressure drop experienced by the system, thus, spherical particles show significant lower pressure drops than amorphous silica gel (Figure S1A), which is expected giving the combination of significantly different particle size as well as the irregular shape and size of silica particles. A larger void fraction is expected to result in a lower pressure drop. Pressure drops are further reduced when using porous particles, potentially allowing for longer reactors before nearing the working pressure limit for the tubing. Figure S1B shows the particle size also affects the pressure drop as expected, with lower particle sizes generating higher pressures. Overall, the results show both solid and porous beads would allow the use of a ~10 m long reactor while still working below the maximum safe working pressure (< 300 psi) demonstrating the ease of scalability of this platform.

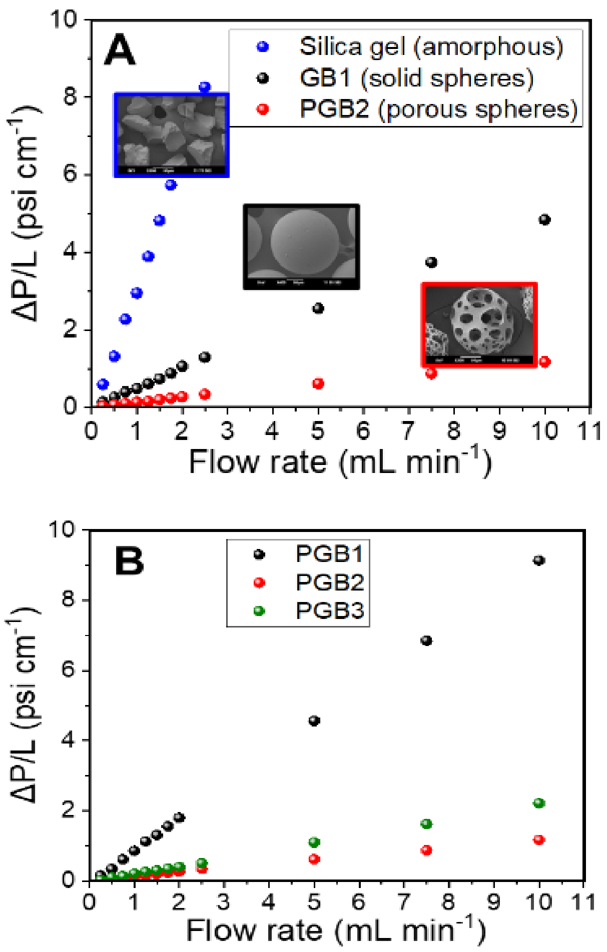

Figure S1. Back pressure experienced as a function of IPA flow rate over a fixed-bed reactor (ID = 0.085 in) for amorphous silica gel, GB1, PGB1, PGB2 and PGB3. The plots compare particle morphology and particle porosity (A), and porous particle size (B). RSD < 1 %.

As catalyst loading requires pre-activation of the particles under either acid or basic conditions, the materials were subjected to these conditions and their chemical resistance was visually evaluated. In the case of the solid borosilicate glass beads (GB1), the surface activation could be completed with Piranha solution resulting in sticky, hydrophilic beads due to the increased concentration of surface Si-OH groups. As the same conditions lead to complete disintegration of the porous beads (PGB1, PGB2 and PGB3), alternative basic conditions (1 M NaOH, aq.) were used to activate these materials or activation step was removed completely.

Pressure drop calculations

The Ergun equation (Eq. 1) describes the pressure drop ($\Delta P/L$) experienced during fluid flow over a fixed bed of particles, of length (L), as a function of the packed bed void fraction (e), the mean particle diameter (d_p), the fluid density (ρ) and fluid viscosity (μ), and the sphericity of the particles (ϕ, equals to 1 for spherical particles).

$$\frac{\Delta P}{L} = \frac{150\mu(1-\varepsilon)^2\mu_0}{\phi\varepsilon^3 d_p^2} + \frac{1.75(1-\varepsilon)\rho\mu_0^2}{\phi\varepsilon^3 d_p} \qquad \text{Eq. 1}$$

Pressure drop experiments

Pressure drop experiments were conducted using a Teledyne Isco syringe pump to both deliver IPA across the packed bed as well as provide a pressure reading via a built-in pressure transducer (zero'd to atmospheric pressure).

Materials characterization

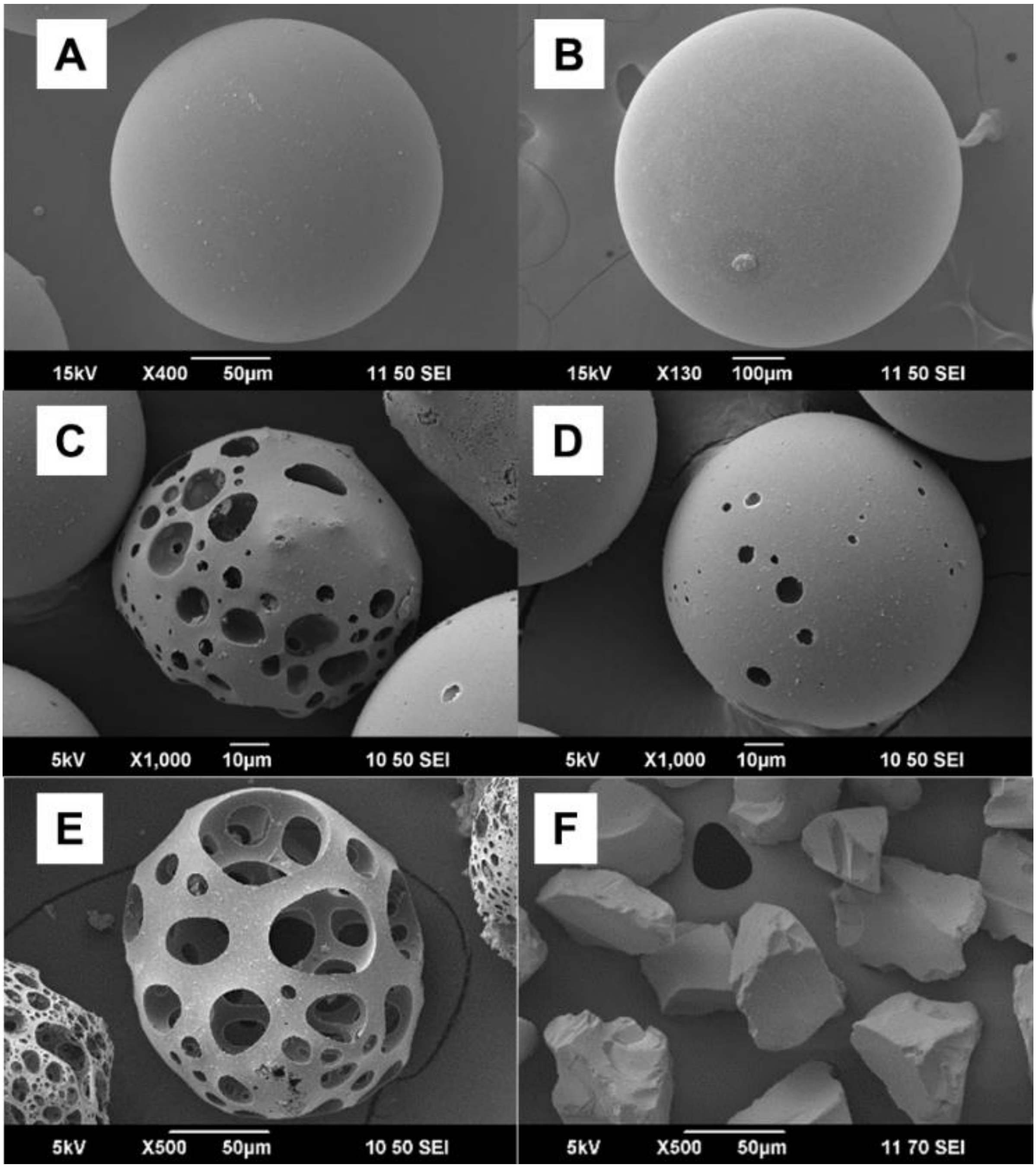

Figure S2. Representative scanning electron microscopy images of A) GB1, B) GB2, C) PGB1, D) PGB2, E) PGB3, and F) silica gel for comparison.

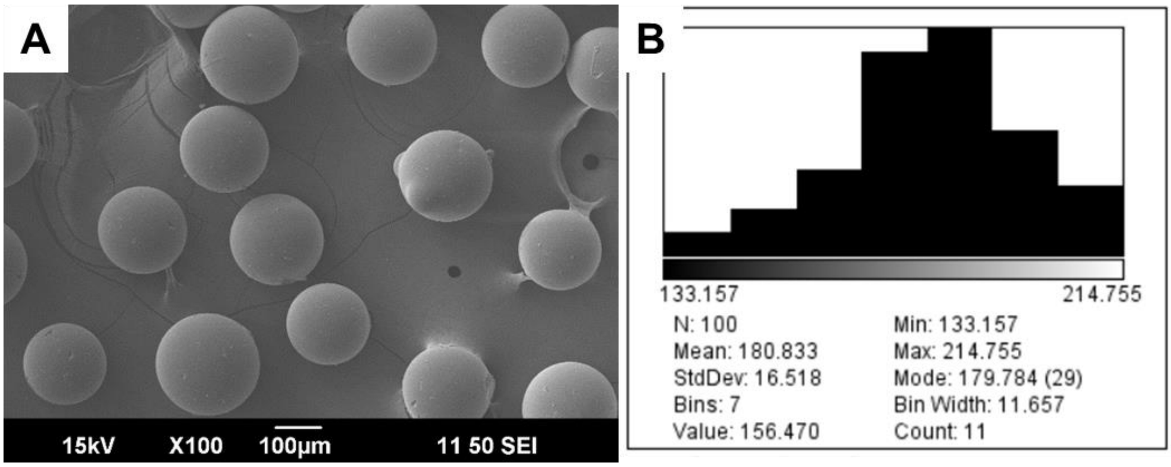

Figure S3. Low magnification SEM image of GB1 (A) and the corresponding particle size distribution (measured using ImageJ) (B).

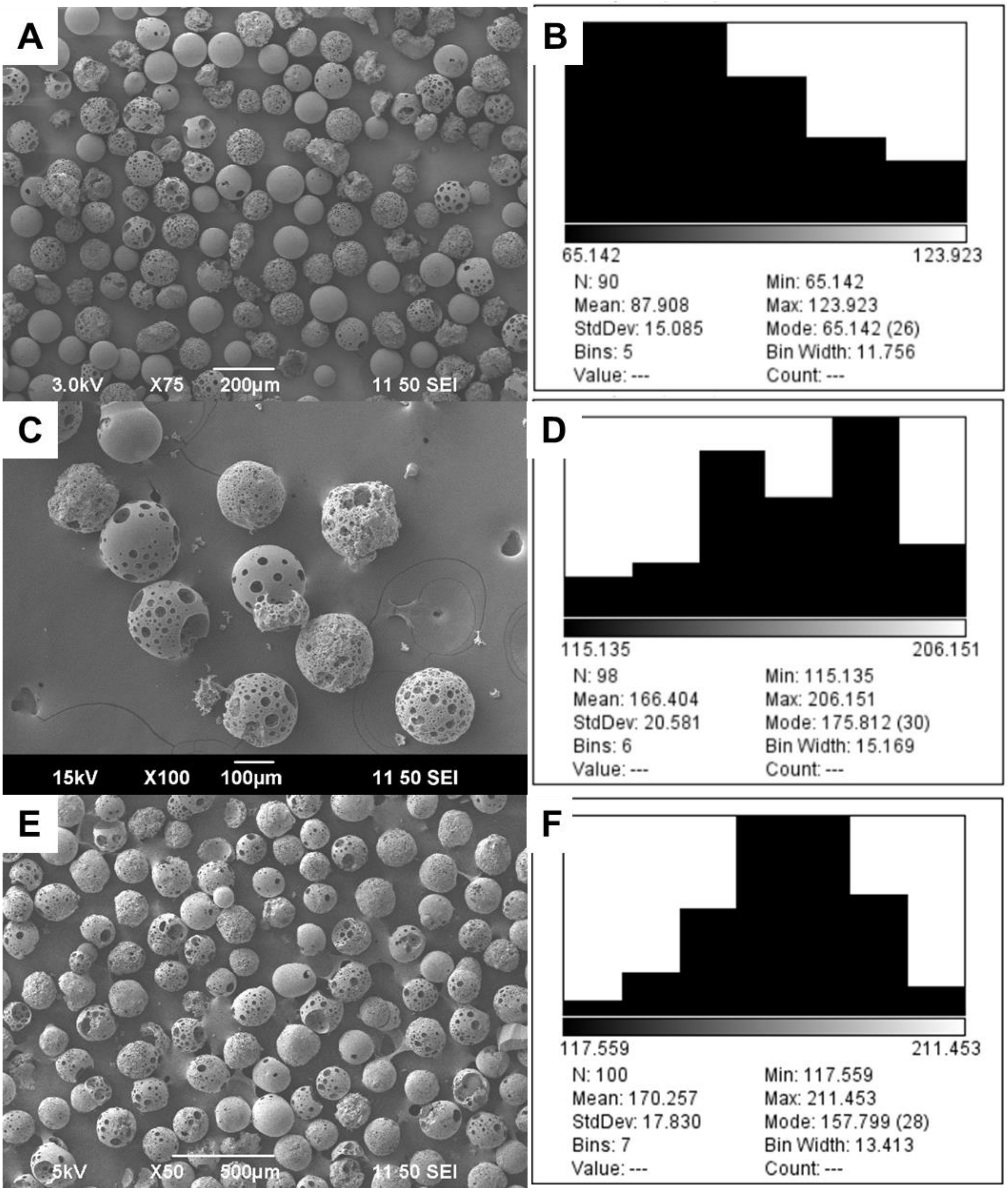

Figure S4. Low magnification SEM images of A) PGB1, C) PGB2, and E) PGB3; and the corresponding particle size distributions B), D), and F).

Photocatalyst preparation

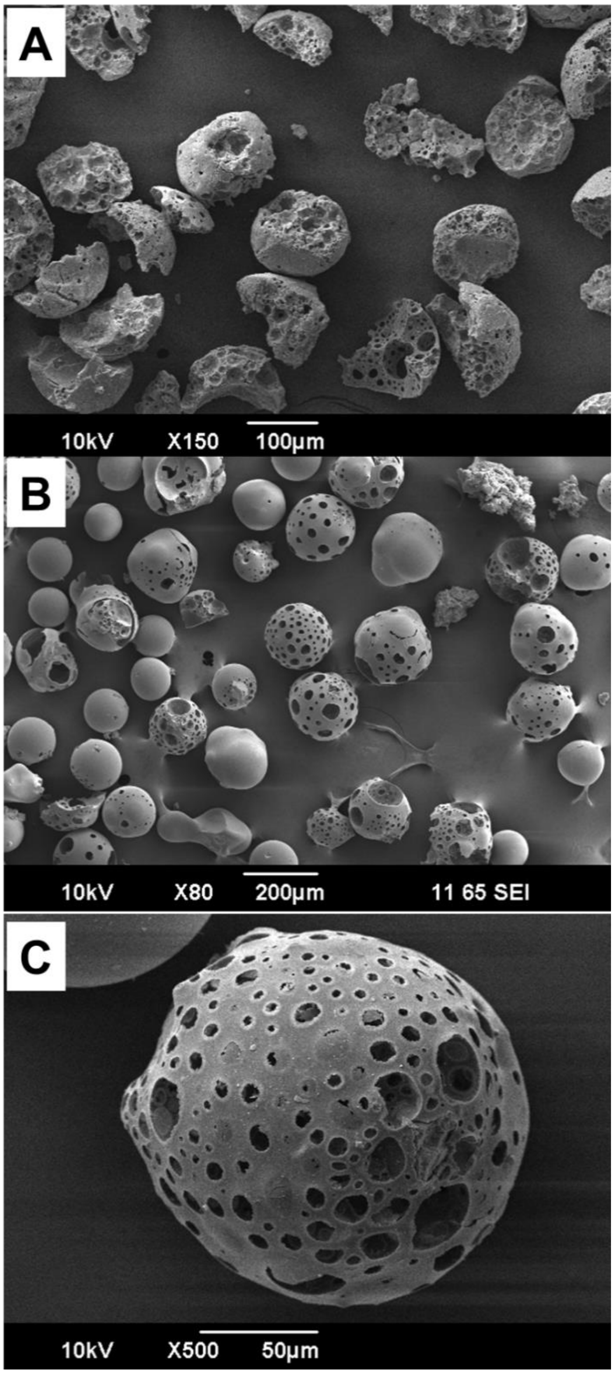

Figure S5. A) Low magnification (x150) SEM image of PDI-PGB3 after functionalisation showing some rupture structures, B) low magnification (x80) SEM image of PDI-PGB3 after functionalisation without an activation stage showing porous structure is retained, and C) medium magnification (x500) SEM image of PDI-PGB3 showing the presence of micron-sized pores and wall-to-wall porosity.

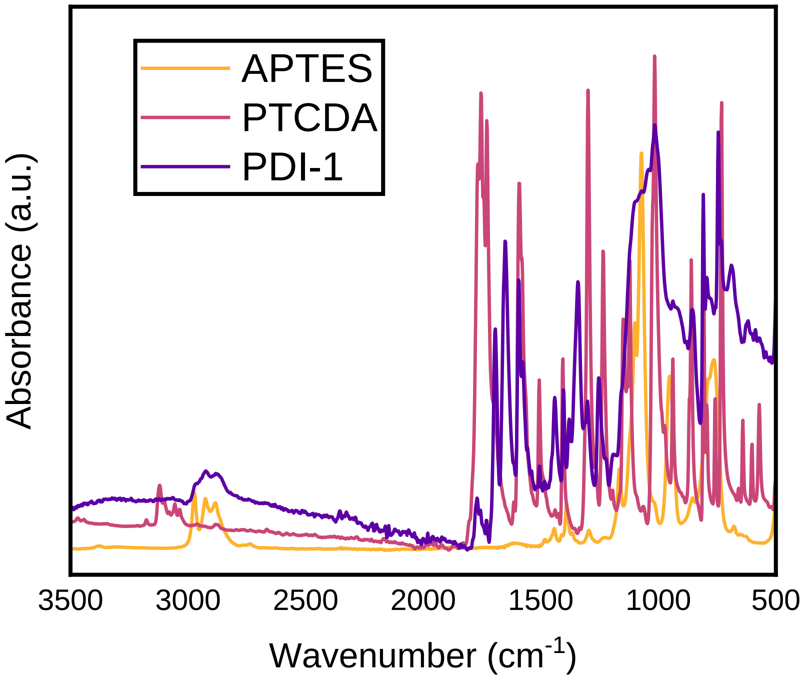

Figure S6. ATR FTIR spectra comparing PTCDA (pink), APTES (amber) and PDI (purple).

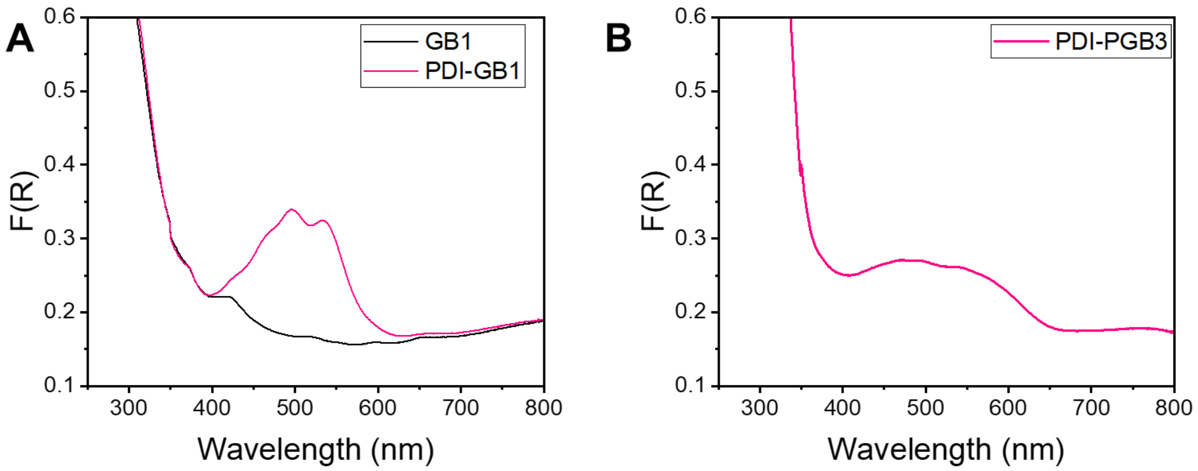

Figure S7. Optical characterisation of PDI-beads: diffuse reflectance (DR) spectra of A) PDI-GB1 and B) PDI-PGB3 beads prepared without activation (largely intact, Figure S5B).

PDI loading

Theoretical PDI loading can be estimated on the bases of the following assumptions: 1) PDI forms a monolayer on the surface of the beads, 2) glass beads total surface area can be calculated using the average particle size, d_{mean}, and assuming beads are perfectly spherical, 3) borosilicate glass density is 2.23 kg L^{-1}, and 4) the footprint of a PDI molecule is ca 1 nm^2. Errors could arise from the improper formation of a monolayer (e.g. multilayer formation), the formation of a polymeric PDI network as well as the presence of non-spherical beads.

Experimental PDI loading was determined by removing the superficial dye layer using an aqueous NaOH solution (0.5 M) and ultra-sonication for 30 mins. Alternatively, the mixture of beads (250 mg) and NaOH (0.5 M, 5 mL) can be stirred at 45 °C for 1 hour, as per published methods[7].

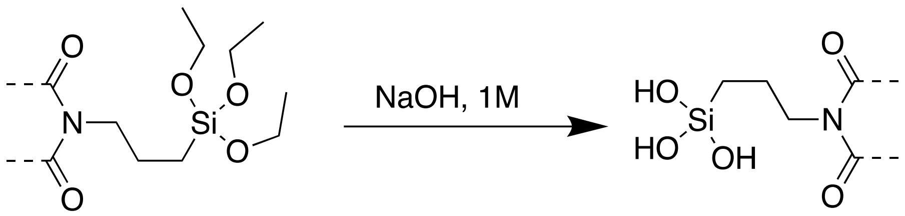

Scheme S1: Hydrolysis of silane from support surface

PDI solutions prepared by hydrolysing the ethoxide groups on the PDI using NaOH lead to a fluorescent solution, which allows for quantification using fluorescence spectroscopy. Note this method is limited and that high PDI loadings on a glass surface can lead to non-fluorescent suspensions of pink particles which assemble into red aggregates over long periods of time.

Table S2. Theoretical PDI loading.

Glass bead	Average diameter (μm)	Surface area/volume ratio	Estimated PDI monolayer coverage (μmol g^{-1})	Experimental PDI loading (μmol g^{-1})
GB1	175	343	0.125	0.195[a]
PGB3	170	353	0.091[b]	*

[a]RSD: 2.7% [b]70% of monolayer surface area as ~30% of bead surface area is lost due to presence of pores. *PDI@PGB3 beads disintegrate upon exposure to dye detachment protocol.

Photochemical reactors

Figure S8 shows the reactors utilised in this work: A thin film photoreactor modelled after the 'PhotoVap' developed by Poliakoff and George[8] and a packed-bed FEP flow reactor[9]. The former was used for batch photochemical experiments and the latter for conducting photochemical reactions in continuous flow.

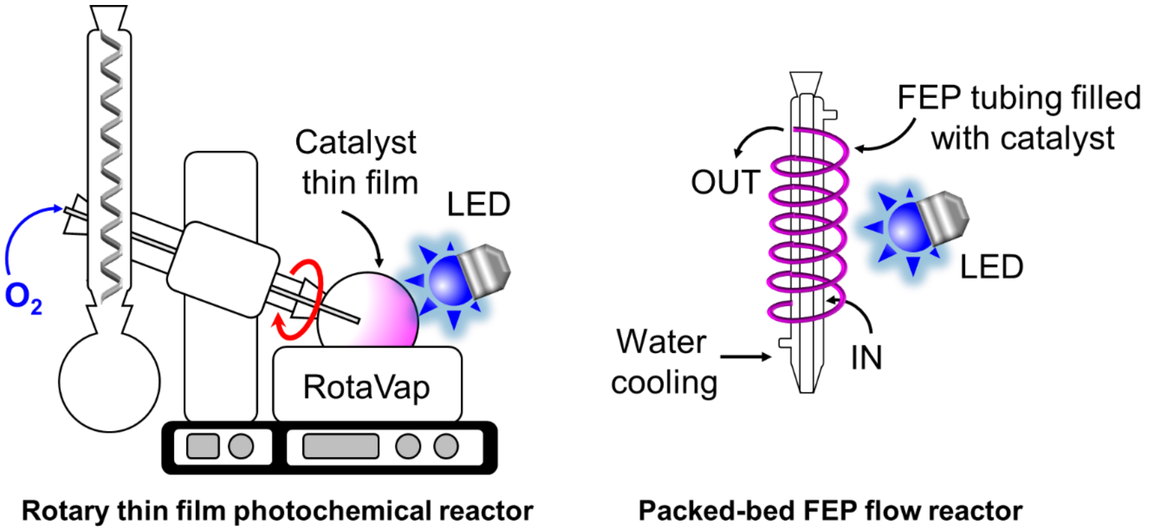

Figure S8. Schematic representation of the photoreactors used in this work (Adapted from ref [8]. Copyright 2018 American Chemical Society).

Batch experiments in the rotary thin film photochemical reactor: A 50 mL round bottom flask is charged with 10 mL O_2 enriched EtOH, starting material and typically 2 g of PDI-beads. Solvent enriched by sparging with O_2 delivered from a balloon for 10 min. The rotary thin film photochemical reactor is sealed to maintain an O_2-rich atmosphere (O_2 delivered from a cylinder). If the rotation is insufficient, the glass beads remain at the bottom of the flask and do not effectively mix with the reaction solvent and substrate. If the rotation is too fast, the beads form a dense band at the centre of the flask. The ideal rotation (175 rpm) causes the generation of a thin film of liquid containing a well dispersed suspension of PDI-beads. In addition to the size, physical properties such as shape and density play an important role in the quality of the suspension. Leaching of PDI was followed by UV-Vis spectroscopy (Figure S10) during five reaction runs performed in this reactor.

Flow experiments in the packed bed FEP flow reactor: FEP tubing (0.125" OD, 0.085" ID, obtained from Cole-Parmer) was used as the reactor body for the packed bed reactor (Figure S9). The beads were loaded into the reactor as a slurry, with 0.5 cm long glass wool end plugs. The filled reactor was wrapped around a jacketed immersion well with water cooling, resulting in a surface temperature of 20 – 30 °C during irradiation (measured externally and with in-line

temperature monitoring). Liquid reagents were introduced to the system using a syringe pump. This was combined with a flow of O_2 (typically 0.010 – 0.1 mL min^{-1}, set using a mass flow controller, MC series, Alicat Scientific) at an IDEX PTFE T-union (0.050" bore diameter) to form a segmented gas-liquid mixture which enters the packed bed of PDI-beads. The reactor was irradiated with a blue LED (459 nm, 1.2 W cm^{-2}) from one side of the coil (reflecting foil placed on the other side to improve light capture). The void fraction was estimated to be 0.4 which significantly reduces the space available for reaction while providing a high catalyst to reactant stoichiometry. For example, a 1.5 m length reactor has an empty volume of 5.5 mL and the loading of beads reduces this to ~2.2 mL. To determine this, it was assumed that the void fraction is comparable to the mass of water required to fill a loaded and dried packed bed via two different experiments. First, a loaded PBR was dried out and left in an oven at 45 ºC overnight, then the mass of the dried PBR was compared with that of a PBR filled with water. This value was also determined by filling a measuring cylinder with a fixed mass of glass beads and adding a quantity of water until the surface of beads is visibly "wetted". Across these two methods, the void fraction was consistently measured to being between 35-40%, comparable with models of hard sphere packing within a cylinder.

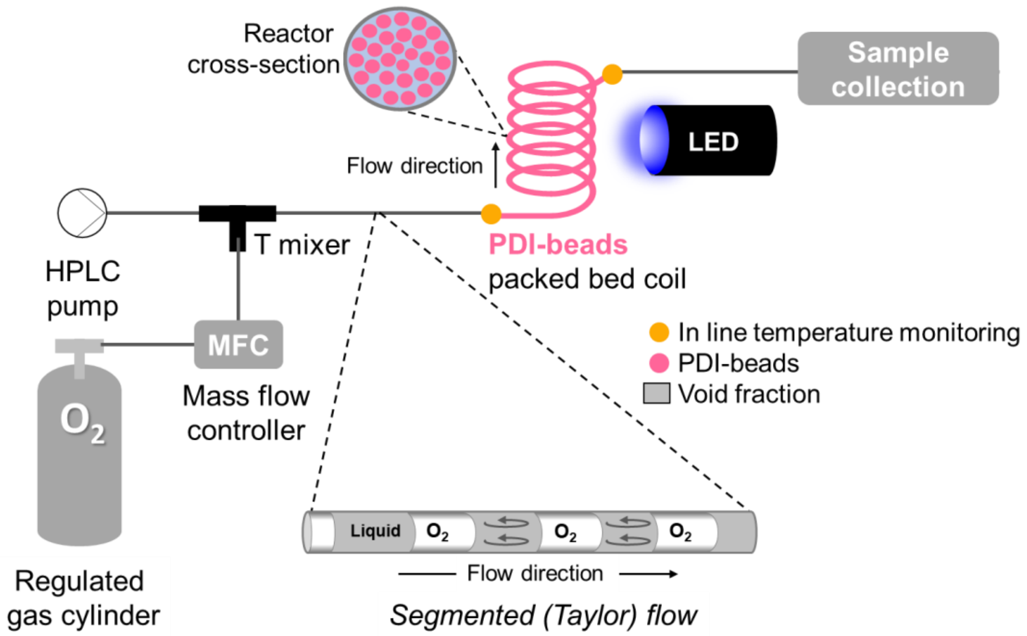

Figure S9. Complete schematic representation of the flow system used in this work, as shown in the scheme the reactor is vertically oriented and the fluids are flown from the bottom of the coil upwards at a slight pitch (corresponding to the pitch of the coil wrapped around the condenser).

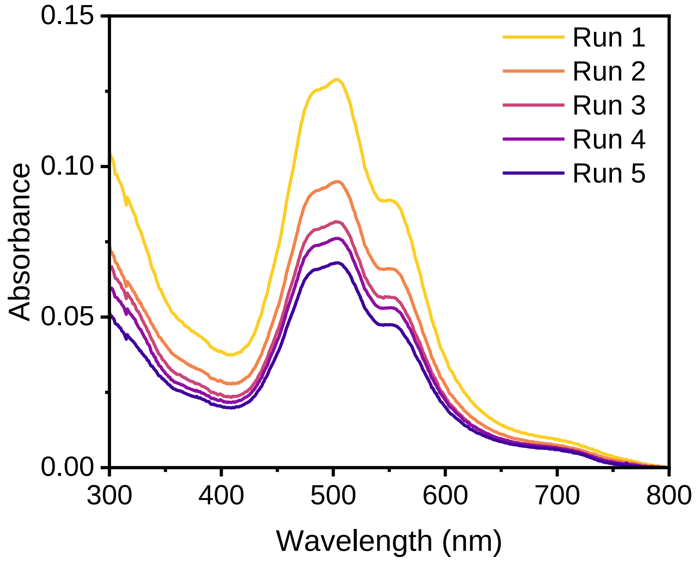

Figure S10. Absorption spectra recorded from each catalytic cycle run under batch conditions. Considering PDI extinction coefficients are typically in the tens of thousands (~40,000 $M^{-1}cm^{-1}$),[10] the estimated amount of PDI loss is <1% of the total PDI loading (assuming a PDI loading of 0.195 μmol g^{-1}). Note the leaching could be due to formation of multiple layers or covalently bound PDI detaching due to particle attrition resulting from mechanical mixing.

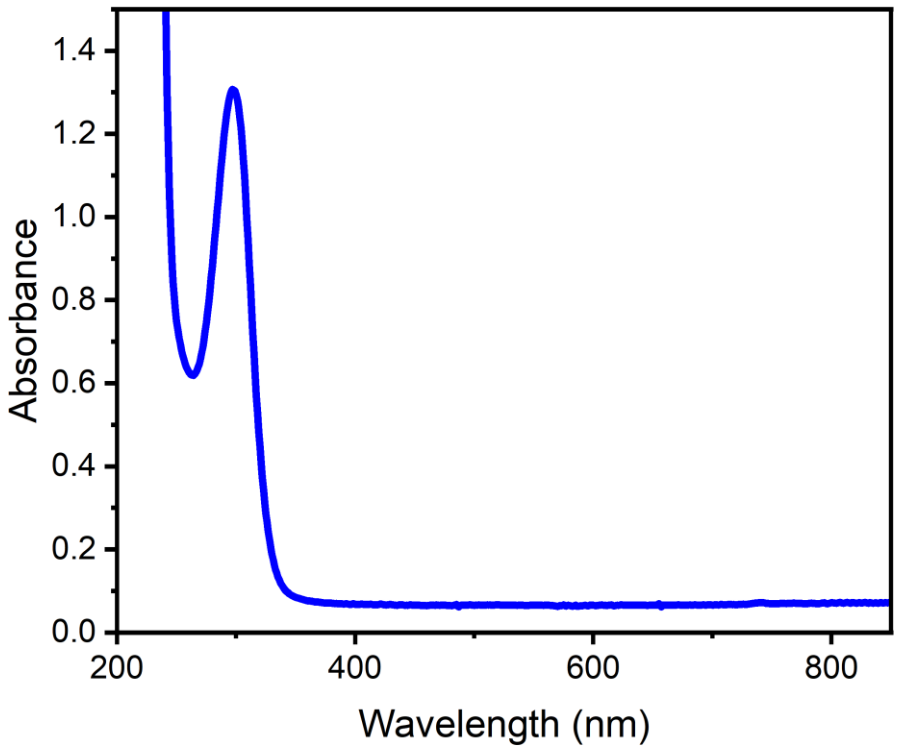

Figure S11. Absorption spectrum recorded from a reaction run under flow conditions. No PDI loss was detected under absorbance (blue) or fluorescence spectroscopy (not shown). The peak centred at ~300 nm corresponds to n-butyl sulfoxide (reaction product).

Residence time distribution analysis

Residence time distribution experiments were completed using an OceanInsight miniature UV/vis spectrometer equipped with an in-line flow cell. A Vapourtec R2 flow system was used to maintain a constant flow (50 μL min^{-1}) of EtOH into the system and a pulse of rose bengal dye (5 mM, 63 μL) was delivered using the built-in 6-way valve. The mean residence time was monitored by collecting time-series absorbance data at λ = 559 nm and processed in Origin (OriginLab) using a 5-point cubic Savitzky-Golay filter.

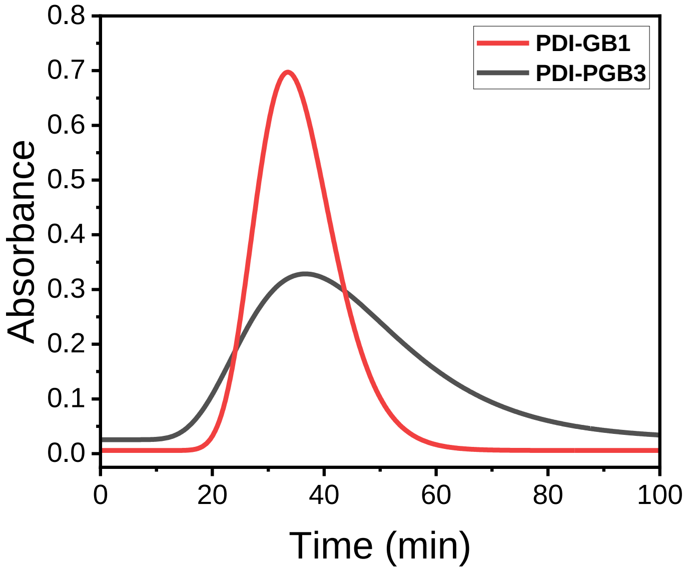

Figure S12. Residence time distribution of a pulse injection through packed beds of PDI-GB1 (red) with mean residence time = 17.8 min and PDI-PGB3 (black) with mean residence time = 28.4 min when using a flow rate of 50 μL min^{-1}.

Turnover number calculations

The turnover number (TON) of the catalyst was calculated using the equations below:

$$TON = \frac{\text{amount of product generated}}{\text{amount of catalyst}}$$

For instance, for the PBR loaded with 30 g of PDI-beads, the amount of PDI was determined as *ca.* 6 µmol. Reactions typically took 3 reactor volumes to reach steady state and were held for another two for sample measurement. A total of 5 reactor volumes of substrate solution were turned over for each compound in this study.

Turnover frequencies (TOF) were only calculated for batch experiments by dividing the TON by the reaction time.

Table S3. Optimisation of flow conditions for photocatalysed Aza-Henry reaction between N-Ph THIQ and nitromethane.

Entry	Cumulative TON	O_2 (mL min^{-1})	Gas/liquid ratio	τ_{res} (min)	Conversion (%)	Yield (%)	Productivity mmol h^{-1}	Productivity mmol h^{-1} L^{-1}
1[a]	0	0.05	1	4	0	n.d	-	-
2	667	0.02	1	11	35	30	0.05	123
3[b]	667	0.74	1	15	42	35	2.33	1061
4	1333	0.056	1	20	40	34	0.17	78
5	1833	0.074	0.5	10	30	25	0.12	57
6	2750	0.074	2	20	60	49	0.49	223
7	4250	0.074	3	22	90	80	1.07	489
8	5639	0.111	3	15	85	75	1.51	687

Reaction conditions: LED: 456 nm working at 0.58 W cm^{-2}. [a]Reactor length (L): 30 cm (entries 1-2) with 10 cm of 0.0125” ID tubing for pre-reaction gas-liquid segmentation. Reactor volume: ~440 µL (entries 1-2). [b]Reactor length: 150 cm (entries 3-8) with 10 cm of 0.0625” ID tubing for pre-reaction gas-liquid segmentation. Reactor volume: 2.2 mL. τ_{res}: space time. Productivity (mmol h^{-1}) = concentration (M) x flow rate (mL min^{-1}) x yield x 60 min h^{-1}. Space-time yield (STY, mmol h^{-1} L^{-1}) = productivity / reactor volume. N-Ar THIQ (0.075 M) in 4:1 MeCN/$MeNO_2$ solvent. Reaction followed using ^{1}H NMR with 2,5-dimethylfuran as an external standard.

Table S4. Optimisation of flow conditions for photo-oxidation of furfural to 5H5F

Entry	[Furfural] (mM)	Cumulative TON	Q (mL min^{-1})	τ_{res} (min)	Conversion (%)	Yield (%)	Productivity mmol h^{-1}	Productivity mmol h^{-1} L^{-1}
1	100	21917	0.22	20	70	65	0.86	200
2	100	25139	0.11	40	90	88	0.58	130
3	200	29472	0.22	20	65	59	1.56	350
4[a]	200	36083	0.11	40	>95	90	1.19	270
5[a]	200	42250	0.22	20	>95	84	2.22	500
6[a,b]	200	48783	0.3	15	>95	89	3.20	730

Reaction conditions: LED: 456 nm working at 0.58 W cm^{-2}. Reactor length (L): 300 cm with 10 cm of a 0.0625“ ID tubing for pre-reaction gas-liquid segmentation. Reactor volume: 4.4 mL. Q: liquid flow rate, τ_{res}: space time. Productivity (mmol h^{-1}) = concentration (M) x flow rate (mL min^{-1}) x yield x 60 min h^{-1}. Space-time yield (STY, mmol h^{-1} L^{-1}) = productivity / reactor volume. Furfural (0.2 M) in MeOH, p-toluenesulfonic acid (0.05 mol% [entries 4-6 only]). Reaction followed using GC with 1,3,5-trimethoxybenzene as an external standard. [a] 0.05 mol% pTSA added to starting mixture. [b] 60 cm 0.0313 in ID pre-reaction segmentation tubing instead of 10 cm 0.0625 in ID.

References

1. Bimetallic magnetic PtPd-nanoparticles as efficient catalyst for PAH removal from liquid media, Zanato, A. F. S.; Silva, V. C.; Lima, D. A.; Jacinto, M. J., *Appl. Nanosci.* **2017,** 7(8), 781.
2. Preparation and characterization of magnetic photocatalyst from the banded iron formation for effective photodegradation of methylene blue under UV and visible illumination, Sanad, M. M. S.; Farahat, M. M.; El-Hout, S. I.; El-Sheikh, S. M., *J. Environ. Chem. Eng.* **2021,** 9(2), 105127.
3. A novel magnetic photocatalyst $Bi_3O_4Cl/SrFe_{12}O_{19}$: Fabrication, characterization and its photocatalytic activity, Wang, H.; Xu, L.; Liu, C.; Jiang, Z.; Feng, Q.; Wu, T.; Wang, R., *Ceram. Int.* **2020,** 46(1), 460.
4. Photocatalysis meets magnetism: Designing magnetically recoverable supports for visible-light photocatalysis, Terra, J. C. S.; Desgranges, A.; Monnereau, C.; Sanchez, E. H.; De Toro, J. A.; Amara, Z.; Moores, A., *ACS App. Mater. Inter.* **2020,** 12(22), 24895.
5. Porous phosphate-based glass microspheres show biocompatibility, tissue infiltration, and osteogenic onset in an ovine bone defect model, McLaren, J. S.; Macri-Pellizzeri, L.; Hossain, K. M. Z.; Patel, U.; Grant, D. M.; Scammell, B. E.; Ahmed, I.; Sottile, V., *ACS App. Mater. Inter.* **2019,** 11(17), 15436.
6. Fluid flow through packed columns, Ergun, S., *Chem. Eng. Prog.* **1952,** 48, 89.
7. Quantification and stability determination of surface amine groups on silica nanoparticles using solution nmr, Kunc, F.; Balhara, V.; Brinkmann, A.; Sun, Y.; Leek, D. M.; Johnston, L. J., *Anal. Chem.* **2018,** 90(22), 13322.
8. UV photovap: Demonstrating how a simple and versatile reactor based on a conventional rotary evaporator can be used for UV photochemistry, Clark, C. A.; Lee, D. S.; Pickering, S. J.; Poliakoff, M.; George, M. W., *Org. Process Res. Dev.* **2018,** 22(5), 595.
9. A practical flow reactor for continuous organic photochemistry, Hook, B. D. A.; Dohle, W.; Hirst, P. R.; Pickworth, M.; Berry, M. B.; Booker-Milburn, K. I., *J. Org. Chem.* **2005,** 70(19), 7558.
10. Synthesis and excited-state photodynamics of perylene−porphyrin dyads. 1. Parallel energy and charge transfer via a diphenylethyne linker, Prathapan, S.; Yang, S. I.; Seth, J.; Miller, M. A.; Bocian, D. F.; Holten, D.; Lindsey, J. S., *J. Phys. Chem. B* **2001,** 105(34), 8237.
